# The C-terminal subunit of artificially truncated human cathepsin B mediates its nuclear targeting and contributes to cell viability

**DOI:** 10.1186/1471-2121-6-16

**Published:** 2005-04-04

**Authors:** Felix Bestvater, Claudia Dallner, Eberhard Spiess

**Affiliations:** 1 Deutsches Krebsforschungszentrum, PO Box 101949, D-69009 Heidelberg, Germany

## Abstract

**Background:**

Splicing variants of human cathepsinB primary transcripts (CB(-2,3)) result in an expression product product which lacks the signal peptide and parts of the propeptide. This naturally truncated Δ^51^CB is thus unable to follow the regular CB processing and sorting pathway. It is addressed to the mitochondria through an activated N-terminal mitochondrial targeting signal instead. Although Δ^51^CB is supposed to be devoid of the typical CB enzymatic activity, it might play a role in malignancies and trigger cell death/apoptosis independent from the function of the regular enzyme. Cytoplasmic presence of the mature CB might occur as a result of lysosomal damage.

**Results:**

We investigated such "aberrant" proteins by artificial CB-GFP chimeras covering various sequence parts in respect to their enzymatic activity, their localization in different cell types, and the effects on the cell viability. Unlike the entire full length CB form, the artificial single chain form was not processed and did not reveal typical enzymatic CB activity during transient overexpression in large cell lung carcinoma cells. Δ^51^CB was found predominantly in mitochondria. In contrast, the shorter artificial CB constructs localized in the cytoplasm, inside the cell nucleus, and in the midbodies of dividing cells. Bleaching experiments revealed both mobile and immobile fractions of these constructs in the nucleus. Nuclear accumulation of artificially truncated CB variants led to disintegration of nuclei, followed by cell death.

**Conclusion:**

We propose that cell death associated with CB is not necessarily triggered by its regular enzymatic activity but alternatively by a yet unknown activity profile of truncated CB. Cytoplasmic CB might be able to enter the cell nucleus. According to a mutational analysis, the part of CB that mediates its nuclear import is a signal patch within its heavy chain domain. The results suggest that besides the N-terminal signal peptide also other CB domains contain patterns which are responsible for a differentiated targeting of the molecule, e.g. to the mitochondria, to the nucleus, or to vesicles. We propose a hierarchy of targeting signals depending on their strength and availability. This implies other possible transport mechanisms besides the usual trafficking via the mannose-6-℗ pathway.

## Background

Lysosomal cysteine peptidases play an important part in intra- and extracellular protein degradation. Their primarily assumed function has changed: they "can no longer be considered as simple garbage disposers" [1], but do also function as key enzymes in cardinal processes of homeostasis and cell demise. This is particularly valid for the ubiquitous peptidase cathepsinB (CB, E.C.3.4.22.1). In higher organisms, this enzyme is present and active in almost all tissue types. For a long time it was therefore considered as an unspecifically degrading peptidase. Research of recent years has brought up specificity [2] and its implication in pathologic processes as arthritis [3] or cancer [4-6]. Furthermore, these investigations have revealed the pivotal role of CB in a number of apoptotic pathways [7-18].

**Figure 1:**
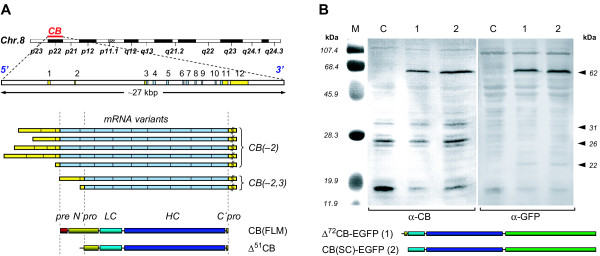
**Expression and processing of CB**. A. Chromosomal location (adapted from NCBI/NIH) and exon-intron organization of the *hCB *gene (top panel, modified from [19]), alternative splicing variants of CB primary transcripts (centre panel, modified from [56]), and domain organization of the entire translation product (bottom panel). Lightened sectors: non-coding exons (5'-UTR and 3'-UTR); darkened sectors: translated CB regions. The transcript population CB(-2) encodes the entire CB sequence, the alternative transcript population CB(-2, 3) leads to the truncated translation product Δ^51^CB which lacks the complete signal sequence (pre) as well as parts of the proregion (N'pro). *In vivo*, the single chain form of the native CB is mainly split into the light and the heavy chain while the C'pro-region is cut off during secretion. B. Processing of intrinsic CB (C, control) and of transiently overexpressed Δ^72^CB-EGFP (1) and CB(SC)-EGFP (2) in LCLC-103H cells (on the left: *α*-CB-Ab; on the right: *α*-GFP-Ab). The immunoblot reveals distinct native CB fragments at similar expression levels of ~31 kDa (single chain form), ~26 kDa (heavy chain), and several isoforms thereof indicated by weaker adjacent bands. Unspecific low molecular mass protein bands below signify degradation products. Additional expression products of ~62 kDa (*α*-CB, *α*-GFP) in the samples from the transfected cells represent entire unprocessed sequences. The Δ^72^CB-EGFP band runs slightly slower than the CB(SC)-EGFP band. Weak bands at ~22 kDa (*α*-GFP) indicate that a small amount of the EGFP tag is removed from the fusion proteins.

The human *CB *gene is composed of 12 or 14 exons [19,20] (Fig 1A, top panel); its promoter is assumed to be regulative [21,22]. The regular mRNA population encodes a 48 kDa polypeptide which contains pre- (signal), pro-, and two functional domains (CB(FLM); Fig 1A, bottom panel). The signal peptide and glycosylated residues target the protein via ER and Golgi into the lysosomes by the mannose-6-℗ pathway. During this process a 31 kDa single chain or 25/5 kDa double chain glycosylated polypeptides are generated. Both forms exhibit enzymatic activity, albeit with different efficiency [19].

A number of mRNA variants may be generated by gene splicing (exon skipping) (Fig 1A, centre panel). The regulation of the splicing process remains unclear. All splicing variants might be expressed concomitantly [23]. They can be subdivided into two subpopulations which result in two distinct translation products [19,20,23,24]. The first species lacks exon2 (CB(-2)), which does not affect translated regions of the entire CB and appears to be a more easily transcribed message [19]. The second one lacks exons2 and 3 (CB(-2,3)). As a result of an additional initiation codon at position 53 within exon4, this message can give rise to the naturally truncated translation product Δ^51^CB (the original term from the literature is used here). Recent publications prove that Δ^51^CB has no regular CB enzymatic activity [25,26]. Such truncations are not exceptional among cysteine peptidases. Recently, there have been discovered other truncated cathepsins like a cathepsinH progeny lacking parts of the signal peptide with unknown functions and differentiated intracellular distribution [27] and a truncated form of cathepsinL devoid of the signal peptide with nuclear targeting and a specific cleaving activity [28]. According to present knowledge and interpretations, the truncated message of CB is linked to pathological findings, in so far as the respective expression product was found more prominently expressed in tumours [24] or arthritic tissues/cells [23]. However, it is not clear whether it promotes malignancies or is a means of defence.

Although the detection of CB outside the lysosomes was primarily considered atypical, its extracellular and plasma membrane associated appearance is now well documented [4,29]. Release of functional CB from lysosomes is connected with apoptosis [13,18]. It is uncertain, whether this can be attributed to a particular fraction of the enzyme.

Earlier on, CB was also found to be associated with the nucleus by immuno- and enzyme cytochemistry [30,31] and by biochemical methods [16,32,33]. The truncated Δ^51^CB was identified in association with the cytoplasmic side of the nuclear envelope [24]. Nuclear fractions of CB could be linked with cell death [7,12,14,33,34]. It remains unclear, whether they represent the mature CB released from the lysosomes or the truncated Δ^51^CB. However, detection methods based on polyclonal antibodies do not discriminate between different variants of CB, just as little as biochemical assays of cellular organelles do between inside and attached (outside) activity. Therefore, the results mentioned above should be considered with care. Both constraints can be overcome by living cell microscopy of genetically labelled samples. Δ^51^CB tagged by GFP appears accumulated in mitochondria; its overexpression provokes nuclear fragmentation and cell death [36]. Mitochondrial localization is not surprising since the first 20 amino acids of the residual proregion contain a mitochondrial targeting signal (MTS) [25]. These results point out a specific role of Δ^51^CB in cell death pathways besides those, for which the lysosome-released canonical forms of the peptidase might be responsible.

The aim of our studies was to characterize cytoplasmic CB forms lacking the ER signal peptide within living cells. Basically, there are two situations in which cytoplasmic CB can occur *in vivo*: (i) as a mature message product resulting from lysosomal leakage [37-40] and (ii) as naturally truncated Δ^51^CB resulting from alternative splicing [19,23-25,36]. We investigated the intracellular transport of various recombinant artificial CB forms in living cells by GFP labelling and by spectroscopic methods. Living cell fluorescence microscopy now offers a wide range of techniques to collect information about cellular processes and will surpass indirect labelling and studies on fixed material. Our findings support a nuclear targeting of CB produced from incomplete messages and they hence partly go together with the data mentioned before [24,25,36]. We could further elucidate the nuclear transport and the stability of nuclear binding by mutational analysis and light microscopy. Finally, we found a link to cell death for the truncated polypeptides synthetically generated from incomplete messages.

## Results

### Posttranslational cleavage and enzymatic activity

Artificially truncated CB constructs such as Δ^72^CB or the single chain form of CB, CB(SC), were obtained from an established human cDNA full-length message (FLM) by PCR, cloned into a eukaryotic expression vector, and tagged by distinct fluorescent protein (FP) variants. These genetic chimeras were then used for mutational analysis, localization, mobility, and functional studies.

Light and heavy chain (LC and HC) are linked by two residues, which are removed *in vivo *during the zymogene processing. Δ^72^CB resulted from an internal *Kpn*I-site upstream the CB(SC) sequence and revealed a fluorescent expression product in cells implying a regular in-frame translation. The expected alternative translation origin lies seven residues before the first codon of CB(SC) at the M^73 ^position. The polypeptide that would result from this message lacks the complete signal peptide and that part of the N-terminal proregion which encodes the MTS. It is the most similar in size to the 21 amino acids longer native Δ^51^CB encoded by the CB(-2,3) transcript [19] in respect to all the other constructs used.

Neither Δ^72^CB-EGFP nor CB(SC)-EGFP was posttranslationally cleaved in LCLC-103H cells during their transient expression as specified by SDS-PAGE and Western Blot analysis (Fig 1B). In addition, only a small fraction of the C-terminal fluorescent protein tag appeared to be cleaved off confirming the high stability of the constructs, which was important for the further studies. Both had similar apparent molecular masses of ~62 kDa. The native CB revealed a typical banding pattern primarily representing the single and the heavy chain forms. The amount of native CB remained unaffected by the transient overexpression of the recombinant products.

Both *in vivo *and *in vitro *studies did not reveal any CB specific enzymatic activity during transient overexpression of CB(SC) variants. In contrast, permanently expressed CB(FLM)-FP was properly processed and exhibited significantly elevated levels of enzymatic activity: In LCLC-103H-cells we measured 250 *μ*EU/(*μ*g protein). The cell clone with the highest CB(FLM)-FP expression revealed 2570 *μ*EU/(*μ*g protein). Nuclei were isolated from this cell clone and non-transfected control cells. We found 1150 and 8.4 *μ*EU/(*μ*g protein), respectively. Microscopy revealed that this activity is located in the nuclear envelope.

### Naturally truncated Δ^51^CB

**Figure 2:**
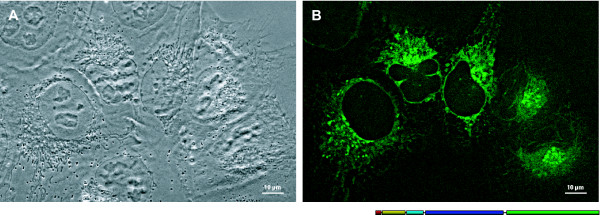
**Intracellular localization of recombinant CB(FLM)**. LCLC-103H cells transiently expressing CB(FLM)-EGFP revealed a strongly developed reticular signal distribution. Besides the ER, the nuclear envelope, the Golgi apparatus, and perinuclear granules were labelled (A: phase contrast; B: fluorescence channel). Obj. 40×/1.30 Oil; post deconvolution.

**Figure 3:**
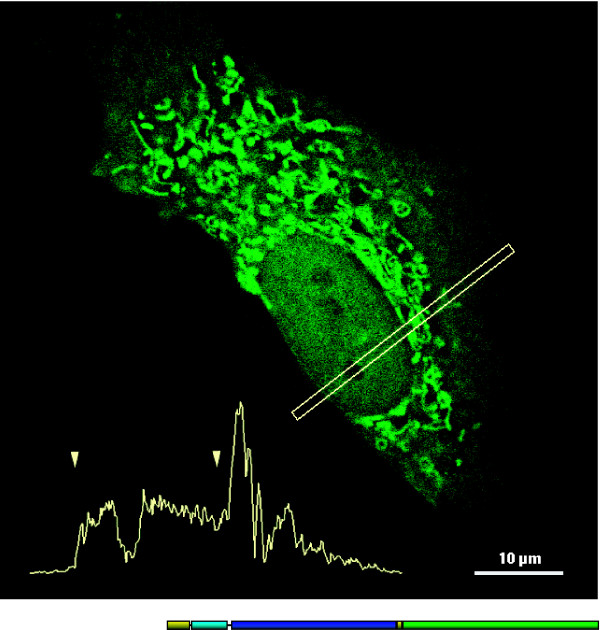
**Intracellular localization of recombinant Δ^51^CB**. LCLC-103H cells with transient Δ^51^CB-EGFP expression revealed an intensive mitochondrial distribution of the fluorescence signals. Beside a slight cytoplasmic background there was also a signal level in the nucleoplasm, which reached ~40% of the mitochondrial fluorescence intensity. The nucleoli were lacking any fluorescence. A profile cut across the cell elucidates the intensity proportions. The arrow tips mark the nuclear boundary. The intensity gap within this region represents a transected nucleolus. For deconvolution, 23 optical sections were used, which have been acquired with a distance of 200 nm. WFM; obj. 63×/1.4 Oil.

The intracellular localization of CB was first studied by a CB(FLM)-EGFP construct (Fig 2). As expected, the expression product was directed into the ER and was mainly found within the Golgi network and the vesicles. Furthermore, we investigated the alternative splicing variant Δ^51^CB tagged by EGFP [25,36]. Expression of this construct in the LCLC-103H cells resulted in strong fluorescence signals consistent with mitochondrial staining (Fig. 3). Their intensity was well above that of the cytosolic background. However, also nuclei exhibited fluorescence, whereas nucleoli were devoid of signal. Quantification of the fluorescence revealed the following order of intensity: mitochondria > nucleus > cytoplasm. The signal ratios are illustrated by an intensity profile of a ROI across an expressing cell (Fig. 3, inset).

### Intracellular localization of the single chain form

A number of established cell lines and freshly isolated endothelial cells were transfected with the chimeric single chain form CB(SC)-EGFP. The transient expression was analysed by fluorescence microscopy the following days. Pure EGFP, which lacks any targeting sequences, was used as control. In all cases, the artificially truncated CB without its signal peptide localized significantly different from the entire CB(FLM)-EGFP construct and Δ^51^CB.

**Figure 4:**
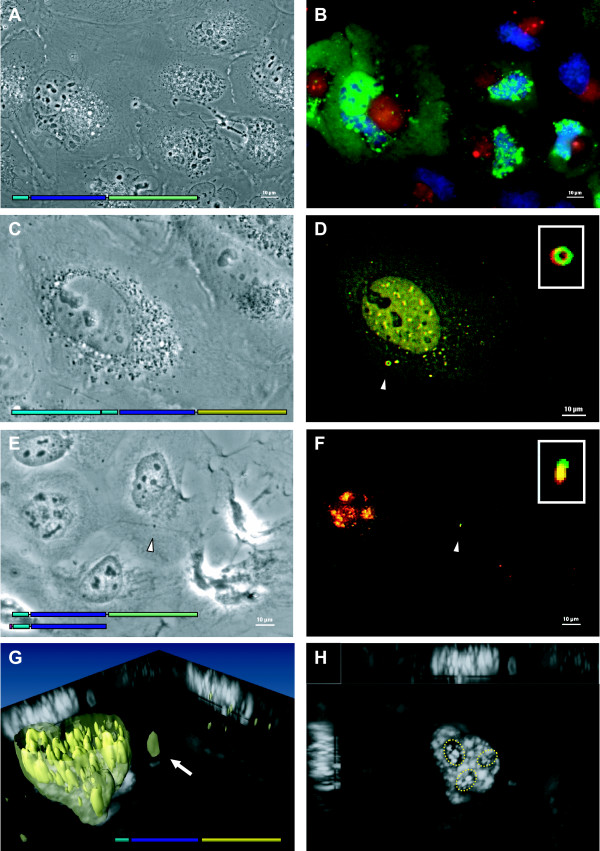
**Intracellular localization of recombinant CB(SC)**. Transient overexpression in LCLC-103H cells visualized by WFM (A-F) or OPM (G, H). A, B. Cells expressing CB(SC)-EGFP (green) were counterstained with Hoechst33342 (blue) and LysoTracker Red (red). Diffuse, vesicular, and granular EGFP fluorescence signals were found in the cytoplasm and highly enriched inside the nucleus sparing out the nucleoli as indicated by the DNA staining. The reticular and vesicular staining of the lysosomal marker adjacent to the nuclear indentation did not overlap with EGFP signals. Obj. 40×/1.30 Oil. Processing of recombinant CB(SC) and influence of the fluorescent protein marker or the total molecule size on its localization were proven by differential tagging (N- or C-terminus, respectively) as well as by means of immunocytochemistry using a N-terminal myc-epitope. C, D. Double-tagged ECFP-CB(SC)-EYFP was distributed mainly in the nucleus analogously to CB(SC), which was marked at its C-terminus only. Accumulates were found within the nucleus and adjacent to it. Fluorescence also appeared in the ring shaped midbody matrix (enlarged region in the upper right corner). Obj. 40×/0.60; processed by deconvolution. E, F. Cells coexpressing myc-CB(SC) and CB(SC)-EGFP were fixed by acetone/methanol and immunostained against myc and GFP. A tight colocalization of both was found (E and F); the constructs were found mainly in the nucleus and stained the midbody (marked by the arrowhead; see inset). Obj. 40×/0.60. G, H. Optical sections of CB(SC)-EYFP expressing cells were subjected to spatial reconstruction (G: 3D-visualisation; H: orthogonal projection). Isosurfaces obtained by arbitrary fluorescence intensity thresholds represent distinct compartments (granules: opaque; nucleus and midbody: transparent). In the given cellular state, only a weak expression in the cytoplasm is found. The main signals arise from granular inclusions within the nucleus including distinct regions inside the nucleoli (marked by dashed lines in H) as well as from enrichment in the midbody (arrow). Obj. 63×/1.32 Oil. ROI: 72 × 59 × 11 *μ*m^3 ^(= 464 × 380 × 25 voxels).

The CB(SC)-EGFP construct appeared in the cytoplasm, but not in the ER or the Golgi of LCLC-103H cells (Fig 4A, B). Apart from that, it was enriched in perinuclear granules and prominently in cell nuclei showing either a homogeneous or a patterned distribution. The nucleoli showed a speckled staining pattern (Fig 4G, H). Fluorescence was also detected in the matrix of the midbody (Fleming-body) which is a persistent remnant of the spindle apparatus of mitotic cells and contains nuclear material among others (Fig 4C–G).

#### Transient transfection of other cell lines than LCLC-103H resulted in similar localization of CB(SC)

HeLa cells and Hep-G2 hepatocytes produced higher numbers of perinuclear granules. Wi-38 fibroblasts showed vesicular structures even in peripheral regions of the cells (pseudopodia), but lower accumulations in the nuclei. The fluorescence distribution was strongly associated with the nucleus in MDCK cells. The localization in the COS-7 cells was more homogeneous in the cytoplasm with no particular accumulations and only low signals in the nucleus. Primary endothelial HDMEC contained intensive, punctual signals in the cytoplasm; nuclei were not involved. Hence, with the exception of HDMEC cells all cell types investigated revealed no principle qualitative differences. The absence of nuclear fluorescence in the primary endothelial cells might depend on a retarded or dysfunctional transport mechanism.

The nuclear accumulation of the CB(SC)-EGFP polypeptide, which is consistent for different cell types from various species, suggests the presence of a nuclear targeting mechanism. We did not find any common nuclear localization signal (NLS) within the CB sequence by appropriate computational analysis (PSORT v6.4, © K. NAKAI, University of Tokyo, Japan). In case of a signal patch, the abundance of nuclear targeting would ask for a specific folding of the polypeptide exposing the patch on its surface. The conformation of the CB(SC)-EGFP polypeptide is difficult to predict. In contrast to CB(FLM)-EGFP, which is folded cotranslationally within the ER, it must be determined by the conditions prevailing in the cytoplasm. To exclude a possible impact of the fluorescent protein sequence on the localization and to prove the integrity of the single chain form (in case of degradation or processing, respectively), further constructs were produced: (i) FP-CB(SC)-FP is flanked by two distinct FPs, (ii) myc-CB(SC)-FP includes a N-terminal myc-Ab epitope (EQKLISEEDL), and (iii) myc-CB(SC) contains this N-terminal myc tag only. FP-CB(SC)-FP showed the same distribution as the single-tagged construct in both fluorescence channels (Fig 4C, D). Application of *α*-myc and *α*-GFP Abs in immunocytochemical experiments to fixed LCLC-103H cells verified a strong colocalization of both signals. The product of myc-CB(SC) colocalized with CB(SC)-EGFP in the nucleus, in cytoplasmic granules, as well as in the midbody (Fig 4E, F). The dual-marker-construct myc-CB(SC)-EGFP revealed the same signal overlap. These results suggest that in most cases (i) neither the type (ii) nor the size of the tag (iii) nor the tagging site do exhibit significant impact onto the structure and the localization of CB(SC). In addition, (iv) degradation or processing of the CB single chain form into light and heavy chains under transient expression conditions is unlikely. This was already exposed by the Western Blot analysis (Fig 1B).

### Nuclear binding studies

The mobility of GFP-tagged CB(SC) was studied by *in vivo *photobleaching experiments. Two-photon laser scanning microscopy (TPM) was applied to register loss of fluorescence through continuous irradiation or fluorescence recovery after photobleaching (FRAP), respectively. TPM confers less damage to the fluorochrome as well as to cells and thus prolongs the effective observation time. For comparative purposes, EGFP and two EGFP chimeras – the ribosomal transcription initiation factor TIF1A and the histone H2A – were taken as controls. Their diffusion and binding characteristics can be deduced from their known localization and functional properties. All controls appear in the nucleus to some extent and represent different types of nuclear binding: EGFP is known to have no binding capacity and to diffuse freely [41]. The transcription factor has a mobile (nucleoplasm) and an immobile (nucleoli) fraction. Histones are tightly bound in the nucleus [42]. Constitutively (H2A-EGFP) or transiently (other constructs) expressing LCLC-103H cells were examined. The obtained serial scans were evaluated in respect to their mean grey values by automated image processing routines.

#### Continuous photobleaching

**Figure 5:**
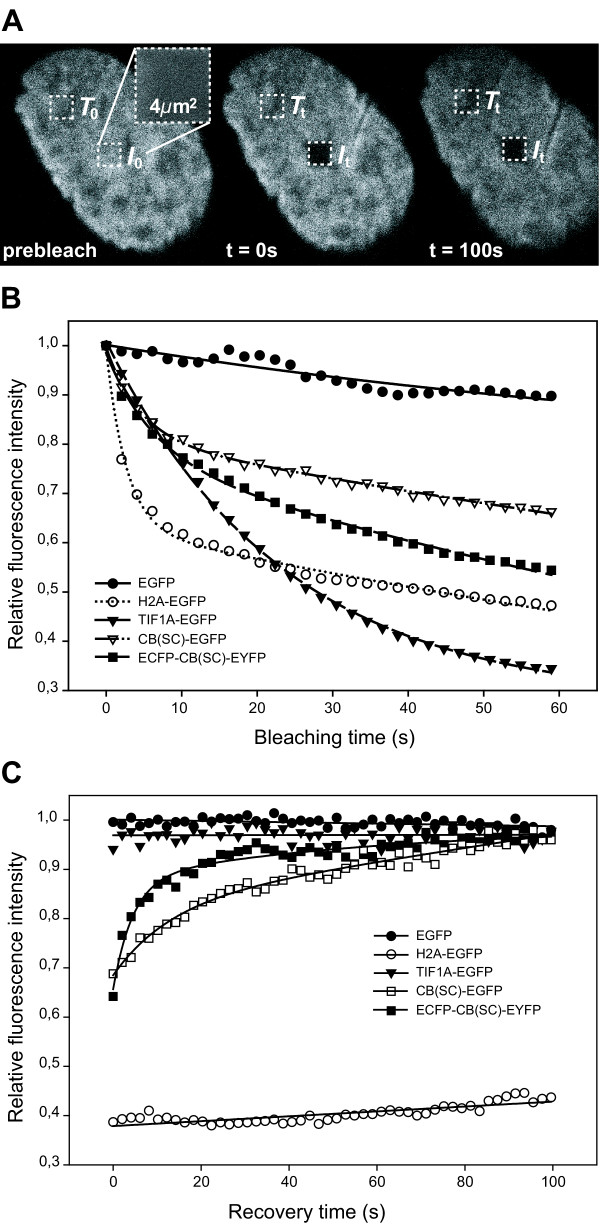
**Nuclear diffusion and binding of recombinant CB(SC)**. Fluorescence was extinguished by TPM within distinct nuclear regions and the diffusion and binding characteristics of GFP-tagged constructs were determined. A. The approach is described by means of the GFP-tagged histone-construct H2A-EGFP. In the continuous photobleaching experiment a 2 × 2 *μ*m^2 ^nuclear region was scanned consecutively and the loss of fluorescence caused by the irradiation was monitored simultaneously (see enlarged section). In the FRAP experiment, a region of same dimensions was bleached by continuous irradiation. A time series was grabbed subsequentially from a larger detail of the nucleus (first and last scan are shown). The fluorescence within the bleached region as well as in an untreated control region was measured and normalized according to equation (1). B. Continuous bleaching curves of CB(SC)-EGFP, ECFP-CB(SC)-EYFP as well as of the control proteins EGFP, H2A-EGFP, and TIF1A-EGFP (n = 2 or 3) are plotted as a function of time. The fitting function is composed of two partial terms and matches the values sufficiently enough and more precise than a simple exponential function. The term meets the fact that there are both bound and freely diffusing fluorochrome labelled fractions. The first subterm describes the bleaching of the bound and the second one the bleaching of the diffusible component. While the graphs for EGFP and TIF1A-EGFP support free diffusion, the H2A-EGFP-population exists mostly in a bound state. Both CB(SC) constructs show an intermediate behaviour which points to bound as well as mobile fractions. C. FRAP curves of the same set of fusion proteins corroborate the findings above.

A nuclear region was illuminated repeatedly and the fluorescence depletion was monitored simultaneously within this ROI in a series of images and evaluated as described in the methods section. The measurements are compiled in (Fig 5B). Decrease of EGFP fluorescence was low, after 1 min of irradiation only 10% of the original intensity have disappeared. This indicates the absence of an immobile fraction. The bleaching characteristics of TIF1A-EGFP were comparable to those of EGFP except of its drastic depletion to a third of its original value. In contrast, the H2A-EGFP curve shows an extremely steep initial decrease – within the first ~10s the equilibrium is reached. This finding suggests that most of the GFP-tagged histone is immobile; a supplementary pool of mobile H2A-EGFP does exist, though. The plots for both CB(SC) constructs (CB(SC)-EGFP and ECFP-CB(SC)-EYFP) show a strong initial loss and lie between the two control constructs EGFP and H2A-EGFP. Hence, we conclude that a significant fraction is not diffusing freely, but is in an associated state.

#### FRAP

The recovery of fluorescence after complete photobleaching within a ROI of the sample was analysed in analogy to the continuous bleaching experiment (Fig 5C). Back diffusion of fluorochrome into the bleached region was monitored continuously. Minor differences in the starting points are inherent to the procedure. The recovery of both EGFP and TIF1A-EGFP intensity was complete within milliseconds. Whereas only mobile EGFP was observed, there was a very small fraction (~3%) of immobile TIF1A-EGFP as indicated by its lower plateau. In contrast, the fluorescence of the tightly bound histone-EGFP chimera did hardly recover and revealed an immobile fraction comprising at least 60% of the material. Again, both CB(SC) variants showed an intermediate behaviour in respect to the controls which confirmed the notion of their binding to nuclear components. The immobile fractions were low and diffusion occurred retarded in both cases. In spite of the higher molecular mass, the double-tagged CB(SC) diffused faster than the single tagged variant. This can be explained by conformational changes in the CB protein caused by the additional N-terminal fluorescent protein, which might affect affinity characteristics. Furthermore, one has to consider the different bleaching characteristics of EGFP (~8% of original intensity) and ECFP/EYFP (~30% of original intensity), which could distort the results.

### Mutational analysis

**Figure 6:**
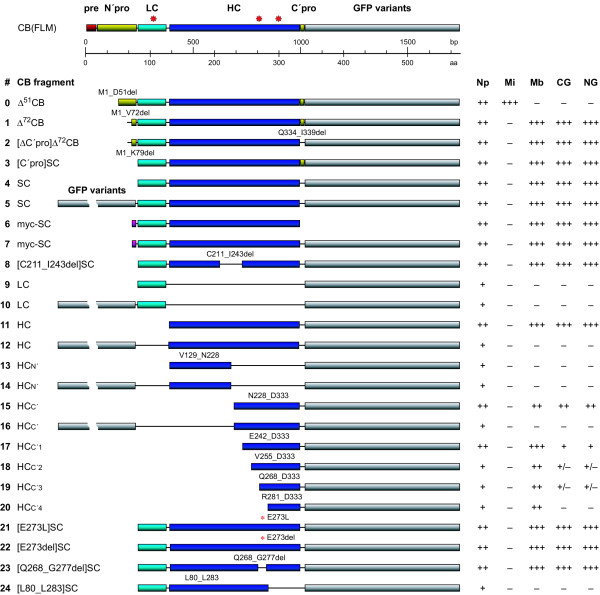
**Mutational analysis**. Besides the recombinant GFP-chimeras of the entire CB zymogene (top panel) and the splicing variant Δ^51^CB (#0), the mutated artificial CB-constructs used in this study (below) and their intracellular localization in LCLC-103H cells (right) are compiled. The active site amino acids in the FLM sequence are indicated by red asterisks. Deleted regions are represented by lines connecting the expressed regions (bars). The N-terminal fluorescent protein tags are displayed in a spliced way. HC: heavy chain; LC: light chain; Δ: deletion; *: site of amino acid exchange; Np: nucleoplasm; Mi: mitochondria; Mb: midbody; CG: cytoplasmic granules; NG: nuclear granules.

**Figure 7:**
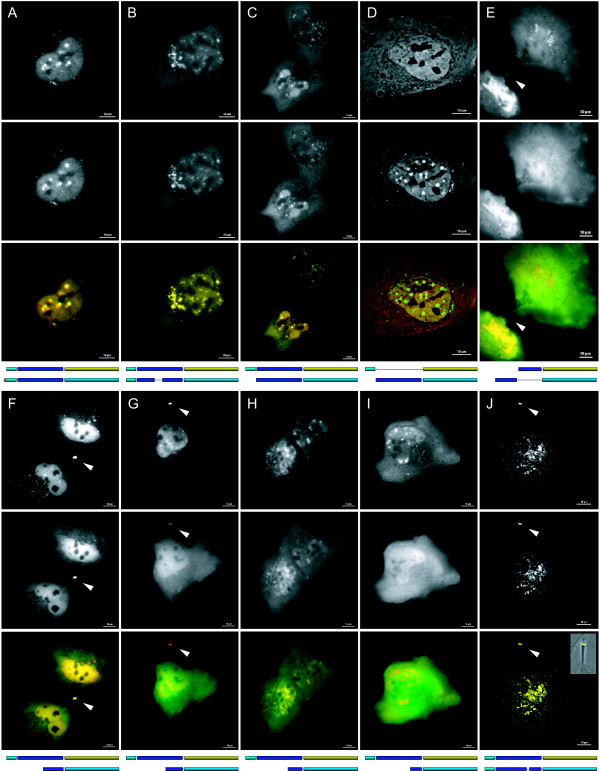
**Localization of mutated CB-FP chimaeras in transiently expressing LCLC-103H cells**. Individual expression with corresponding constructs (above: EYFP-tagged; below: ECFP-tagged) and false colour superposition of both fluorescence channels (YFP: red; CFP: green) are shown. Double-transfection of Δ^72^CB (A) or CB([C211_I243del]SC) (B) with CB(SC), respectively, resulted in a complete colocalization of both constructs. The heavy chain domain still colocalized with the single form to a large extent (C). Compared with the heavy chain, the light chain of CB was distributed homogeneously throughout the cell as pure GFP (D). In contrast to the CB(HC_N'_) constructs, CB(HC_C'_) expressed hotspots around the Golgi and inside the nucleus and stained the midbody (E). Transient coexpression of CB(SC) and its increasingly truncated C-terminal fragments (F-J) revealed decreased nuclear fluorescence with smaller fragment size. Already in case of CB(HC_C'1_) (F), the tendency to accumulate was reduced considerably and the accumulations disappeared completely in case of CB(HC_C'4_) transfection (I). The latter showed an almost complete homogeneous signal distribution without any accumulation in the nucleus, the granules, or the midbody, respectively. The elimination of 9 residues in the C-terminal part of CB (Q268_G277del) had no significant influence on the localization; the construct still colocalized completely with CB(SC) (J; the inset is a superposition of the midbody fluorescence with the corresponding phase contrast image). A-C, G-I. OPM; *λ*_Ex._(ECFP): 430 nm; *λ*_Ex._(EYFP): 500 nm; obj. 60×/1.2 Plan Apo water. D-F, J. WFM, in part after deconvolution (D, J); obj. 63×/1.25 Oil (D, F, J); obj. 40×/0.60 (E); bars 10 *μ*m.

The accumulation of CB(SC) in the nucleus was fatal to the cells as will be shown later. It is therefore interesting to find out which parts of the protein are involved in the nuclear localization. We tackled this subject by a genetic mutational analysis of the single chain CB, CB(SC) (Fig 6). For comparative localization studies, the mutated constructs were tagged by distinct GFP variants, which differ in spectral characteristics (EGFP, EYFP, ECFP, and EBFP). The number and the site of tagging were also varied. The findings are compiled in Fig 6; Figs 3 and 7 illustrate the intracellular localization of the most relevant examples.

A construct encoding the natural Δ^51^CB (#0) localized to mitochondria and nuclei. Two constructs originate from the sequence inherent restriction sites: A *Kpn*I deletion led to (i) a seven residues longer construct in respect to the CB(SC) (#3, 4) and contained parts of the N-terminal propeptide (Δ^72^CB, #1, 2). (ii) The *Bgl*II-generated mutant CB([C211_I243del]SC) (#8) lacked 33 residues, which contain a possible disulfide bridge. In the intact polypeptide, this sequence connects the two globular units of the heavy chain. Both mutants localized in cells exactly as CB(SC). The deletion of the 6 residues long C-terminal propeptide in Δ^72^CB (compare #1 and #2) did not exhibit any influence onto the signal distribution. This corresponds to other observations [36]. As constructs containing or lacking the C-terminal propeptide localized identically, we conclude that the chimeras were not processed at this site and that these results are reliable.

In the absence of a canonical NLS, one might suppose that conformation plays the essential part for the localization of artificially truncated CB. Our efforts were therefore concentrated on globular protein domains the destruction or deletion of which should less affect the folding of the remaining polypeptide. Two GFP-tagged constructs, which correspond to the heavy and the light chain of CB, were produced. While both CB(LC) constructs (#9, 10) revealed a diffuse distribution in LCLC-103H cells comparable to that of pure EGFP, the single-tagged CB(HC) construct (#11) was distributed identically to CB(SC) in the cytoplasm, the nucleoplasm, the granules, and the midbody. This leads to the assumption that the nuclear localization patch really exists and that it is confined within the heavy chain. Double-tagged constructs led to a less differentiated signal distribution in some cases (e.g. #12, 16).

**Figure 8:**
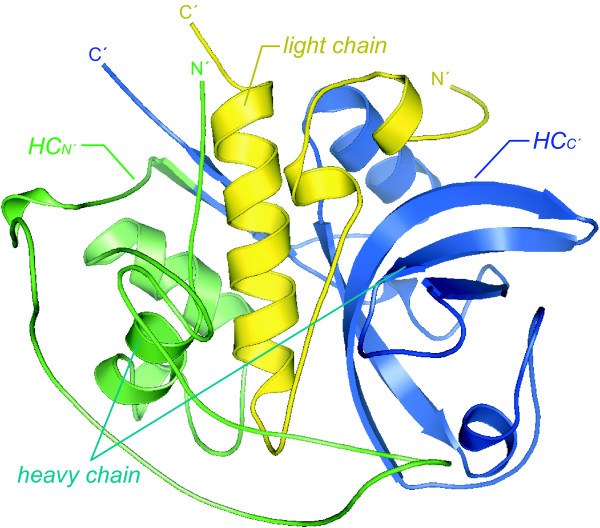
**Spatial representation of the mutated sections**. The positions of key fragments are indicated within the native mature CB protein. These are the globular domains of the light chain, the heavy chain, and two subunits CB(HC_N'_) and CB(HC_C'_). The figure serves for illustrational purposes only and does not represent the authentic conformation of the fragments.

To further narrow down the region of interest, the heavy chain sequence was subdivided into two parts of comparable size CB(HC_N'_) (V129_N228; #13, 14) and CB(HC_C'_) (N228_D333; #15, 16), which encode globular units in the native protein (Fig 8). CB(HC_N'_) was found in the cytoplasm and in low amounts in the nucleus. In contrast, CB(HC_C'_) was highly enriched in the nucleoplasm including granules and also in cytoplasmic granules as well as in the midbodies.

Consequently, the CB(HC_C'_) sequence was reduced equidistantly towards its C-terminus and the following constructs were obtained: CB(HC_C'1_) (E242_D333; #17), CB(HC_C'2_) (V255_D333; #18), CB(HC_C'3_) (Q268_D333; #19), CB(HC_C'4_) (R281_D333; #20). With lower fragment size, the constructs were located more and more homogeneously within the cytoplasm and the nucleus, the typical granules no longer appeared and fewer signals were enriched in the midbody. Finally, the only 52 residues long CB(HC_C'4_) fragment appeared almost as diffuse as EGFP.

The exposed and highly mobile residue E^273^, which is located within the restricted region, raised the question whether it is possibly involved in the nuclear localization of CB. We proved this suspicion by specific point mutations: In CB([E273L]SC) (#21), the acidic and polar glutamic acid is exchanged against the neutral and non-polar leucine; in CB([E273del]SC) (#22), the glutamic acid is eliminated. Finally, in CB([Q268_G277del]SC) (#23), the surrounding residues forming a loop are deleted completely. Surprisingly, neither the point mutation nor the point deletion nor the excision of the five residues to each side of E^273 ^produced a significant change in the localization. In contrast, the elimination of the adjacent C-terminal region in CB([L80_L283]SC) (#24) resulted in a considerable decrease of nuclear signal.

These mutational studies suggest that a region within the C-terminal sequence N228_D333 has a great impact on the nuclear localization of CB(SC). The results favour the assumption that an epitope is generated by a spatial arrangement of the respective, yet not defined residues.

### Mechanisms of nuclear import

Besides an active import of the CB(SC) polypeptide into the nucleus, a passive transport by diffusion processes combined with retention of the molecule inside the nucleus and caused by affinity to nuclear components, has to be considered. In general, proteins larger than 60 kDa cannot pass the nuclear envelope by mere diffusion. To exclude the possibility of passive diffusion, we used a double-tagged construct (FP-CB(SC)-FP; #5) such increasing the size to 84 kDa, which is well above the exclusion limit.

In spite of their increased size, the double-tagged constructs revealed a nuclear localization comparable to that of their single-tagged analogues (Fig 4C, D). Because of the close neighbourhood of both fluorochromes, fluorescence resonance energy transfer (FRET) is possible when appropriate fluorochromes are used. Indeed, using the combination ECFP-EYFP a FRET effect was observed. As the donor molecule is never completely extinguished, the transfer appears to be incomplete, which is not unusual under the given experimental conditions [43]. FRET would become unlikely if the heavy and the light chain of CB(SC) were segregated by processing or general proteolysis. Therefore, the FRET experiment is a further proof that the single chain form keeps intact and that the entire fusion protein is transported into the nucleus.

We conclude that the underlying mechanism depends on an active transport. Further, these results clearly point out a directed nuclear transport and retention mechanism linked to the structure of the CB(SC) polypeptide. In advanced expression states, punctuate signals were observed, which were carried from the tips of the pseudopodia towards the Golgi area in a directed way and which penetrated the nuclear membrane without a noticeable delay [see Additional file 1].

### Induction of cell death

**Figure 9:**
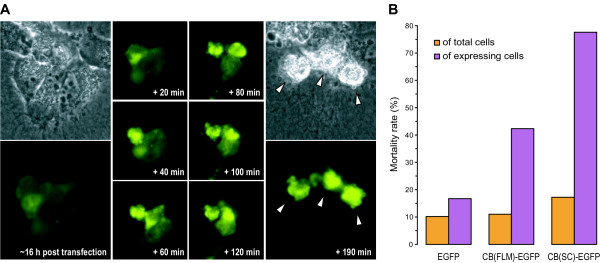
**CB induced cell death**. A. LCLC-103H cells were transiently transfected with CB(SC)-EYFP and the temporal expression course was continuously (Δt = 2 min) monitored beginning with early signs of fluorescence ~12 h post transfection. The process always followed the same scheme: Initially the yet weak fluorescence is distributed equally in the cytoplasm and in the nucleus. Then, fluorescent granules are formed (partly in the pseudopodia) and transported towards the nucleus passing the Golgi region. Granulation goes ahead with rapidly increasing fluorescence intensity in the nucleus. Finally, the Golgi apparatus disappears and the cell collapses within a few minutes while bubbling. Selected images taken by indicated points of a time-lapse experiment document these events. B. Mortality rates of LCLC-103 H cells transiently expressing CB(SC)-EGFP and control constructs (EGFP, CB(FLM)-EGFP) were determined by propidium iodide staining followed by FACS analysis. To meet falsifications of results that are caused by variable transfection rates (22–63%) the absolute mortality values were normalized to the respective transfection efficiency. Dead cells in consequence of the toxicity of the transfection agent and false positives attributable to autofluorescence were taken into account by subtraction of the respective control values: The number of dead cells (propidium iodide channel) amounted to ~13% in case of mock-transfected control cells; 2% of total were false positive "transfectants" (GFP channel). In comparison to CB(FLM)-EGFP, the toxicity of the CB(SC)-construct was almost twice as high (~78%).

LCLC-103H cells permanently expressing CB(FLM) chimeras were easy to clone. In contrast, cells transfected with CB(SC)-FP proved to be short-lived and all attempts to establish cell clones with a typical CB(SC)-FP expression failed. We therefore assume that this construct has the ability to provoke cell death. We proved whether it localizes in a time-dependent manner and monitored the process of cell death in time-lapse experiments (Fig 9A). Cell death followed a strict scheme which can be described in morphological terms over time starting as early as 12 h post transfection and finishing about 6 h later: (i) nuclear accumulation throughout rising expression level, (ii) formation of cytoplasmic and nuclear granules (around the Golgi and within the nucleus mostly), (iii) directed transport of granules to the Golgi and the nucleus, (iv) disintegration of these organelles followed by (v) rounding up and detachment of the cells from the support, (vi) stationary motility of the cell and cellular collapse [see Additional file 2]. Interestingly, cells which were transfected with FP-CB(HC_C'_)-FP also died ~3–4 h after apparent expression. This took place in a different manner exhibiting membrane blebbing and cell swelling followed by bursting of the cell.

We quantified cell death by propidium iodide staining and fax analysis 16 h post transfection and determined mortality rates for populations transfected with EGFP, CB(FLM)-EGFP, and CB(SC)-EGFP (Fig 9B). The mortality rate of mock-transfected cell populations was below 20%. Cells expressing the EGFP control only revealed mortality rates of ~18%. On the contrary, ~43% of the CB(FLM)-EGFP expressing cells and ~78% of the cells transfected with CB(SC)-EGFP died at that time. The latter population did not recover in the further time course and only a few cells with a very low expression level or aberrant localization survived.

Indications for apoptosis were proved. In some cases, nuclear fragmentation and membrane blebbing were observed. A well-defined evidence of apoptosis in respect to disintegration of the plasma membrane (Annexin V/propidium iodide assay) or DNA fragmentation (DNA ladder) could not be produced. Nevertheless, one should not exclude apoptosis in favour of necrosis from further considerations.

## Discussion

Over the last years, the perception about the functions of CB has changed considerably. Recently found evidences assign to this 'lysosomal peptidase' key positions in cardinal processes also outside the lysosomes, like in apoptosis or cancer. Technical advances in microscopy and the development of stable chemical and genetic markers for organelles and molecules now facilitate powerful and direct *in vivo *approaches. They permit not only the localization of certain proteins but also the investigation of their intracellular transport, their interaction with other proteins, and their enzymatic activities, as well as the study of the cellular response in respect to overexpression or silencing of specific proteins.

*In vivo*, both normal tissues and especially tumours contain a population of truncated CB, which can be traced to alternative splicing (Fig 1A). Since expression and transport of CB are frequently altered in transformed and malignant cells as well as in cells undergoing apoptotic processes, we gave our attention to the investigation of such CB aberrations. For this purpose, we have labelled several recombinant CB forms by fluorescent proteins and subjected them to living cell imaging by advanced digital microscopy techniques.

### Truncated cathepsin B forms

The naturally truncated Δ^51^CB lacks the complete signal sequence as well as parts of the N-terminal proregion. This product is barred from entering the ER, and thus from further processing and transport by the mannose-6-℗ pathway. However, the residual propeptide contains a MTS which becomes efficient and directs the predominant amount of the respective product to mitochondria [25]. Our own observations confirm this finding. Δ^51^CB is expressed both *in vitro *and *in vivo *as entire 35 kDa product [24]. This corresponds to our findings of further truncated artificial CB sequences irrespective of their size or tagging with markers: all constructs remained intact and they were not cleaved posttranslationally. Earlier assumptions [24] about the possible CB-specific enzymatic activity of Δ^51^CB were recently questioned [25,26].

Obviously, the propeptide is indispensable for proper *in vivo*-folding of the mature enzyme with the typical CB activity. Interestingly, a splicing variant of cathepsin L devoid of the signal peptide also appears associated with the nucleus and exhibits a specific cleavage activity [28]. Therefore, one should take into consideration that the truncated form(s) of CB might have cleaving characteristics, which do not become evident in the standard assays. In this report, we prove that neither the completeness of the sequence nor the CB specific enzymatic activity is relevant to the observed nuclear accumulation and induction of cell death.

### Nuclear localization

Unlike CB(FLM), which is targeted to the lysosomes via ER and Golgi and partly secreted into the extracellular medium, the cytosol-expressed Δ^51^CB is mainly addressed to the mitochondria [25]. Our own experiments with the same construct confirm these findings. However, deduced from our measurements, a non-negligible fraction of the expression product can also be found in the nucleoplasm. Inspection of the published data [25] does not contradict our findings. Nuclear fluorescence cannot arise from unspecific decay or cleavage products inasmuch as double-tagged constructs reveal similar results as the single-tagged ones indicating the integrity of the constructs. Further, we propose a targeting signal downstream of the MTS which alternatively may direct CB and derivatives thereof into the nucleus. Obviously, a hierarchy of signals encoded within the CB polypeptide determines its intracellular distribution pattern. The signal peptide and the glycosylation sites are decisive for lysosomal targeting of the FLM-product. The signal peptide and the propeptide containing the MTS are removed during the maturation process. Thus, the nuclear targeting signal might become active after release of the enzyme from lysosomes into the cytosol. In case of the truncated Δ^51^CB, the MTS is predominant, whereas for the artificially truncated CB forms the nuclear targeting signal is characteristic. In the past, CB was also found in cell nuclei of tumour cells and normal tissues [31,44,45], but until now there are almost no indications to a potential transport mechanism or a specific function. Especially in apoptotic processes, CB and other CB-like peptidases were detected also in the cell nucleus [8,32]. However, these studies still miss a thorough scrutiny for the nuclear localization.

Here, artificially truncated CB-GFP chimaeras were used, from which the Δ^72^CB-construct came closest to the splicing variant Δ^51^CB in respect to its size. However, it was devoid of the functional sequence present in Δ^51^CB that encodes the N-terminal MTS. Not only Δ^72^CB but also the slightly shorter CB(SC) and other considerably shorter CB fragments were first expressed cytoplasmically as expected. In the sequel, they were enriched within granular structures, which were not consistent with lysosomes or mitochondria as might be supposed. Furthermore, these polypeptides were clearly proved in the nucleoplasm of several cell types. Frequently, nucleoli showed discrete regions of labelling. In contrast to the nucleus, which can be entered by both active and passive transport, the nucleoli are addressed exclusively by interaction with nucleolar building blocks [46]. Immunocytochemistry of CB(SC), which was tagged by a myc-epitope, confirms the results of the GFP-tagging. Though, a slightly higher reticular signal distribution was observed. In addition, this proves that in spite of its size, the fluorescent protein does not sufficiently affect the affinity of CB polypeptides to the respective localization sites.

Based on these results, the capacity of cathepsins particularly in the context of nuclear localization has to be reconsidered [47].

### Mutational analysis

There are no clues to an already known NLS in CB according to literature and to our own computational analysis. The findings suggest that the complex differential distribution of artificially truncated CB might depend on distinct targeting signals. To identify the region(s) of a potential nuclear localization signal sequence or a signal patch, respectively, a number of mutated GFP-tagged constructs were produced. Despite the elimination of extensive sequence regions, partly including potential stabilizing elements such as disulfide bridges – e.g. in CB([C211_I243del]SC) –, the specific localization persisted to a high degree. The participation of the CB light chain in this sorting procedure was excluded. The heavy chain determines the nuclear localization only; the region with the highest impact on the specific localization could be narrowed down to its C-terminal subunit. Although constructs smaller than CB(C'1) did not reveal unequivocal results, the smallest of them, CB(C'4), was not targeted specifically. The assumption that the nuclear affinity essentially depends on the prominent acidic and polar surface residue E^273^, which is found within the relevant region, could not be proved by several specific mutations. Excision of the differential part of CB(C'3) and CB(C'4) did also not affect the localization. The deletion did not imply adjacent residues around the active site H^278 ^in CB(C'4), which also might be important. Hence, the results do not support the existence of a linear signal sequence. Rather a composed signal patch is likely, which evolves from the three-dimensional conformation of the polypeptide. In contrast to linear signals, such signal patches are difficult to identify exactly.

The appearance of CB(SC) and other artificially truncated constructs in the midbody is striking (Fig 4C–G). According to a recent study [48], midbodies have a complex composition. However, only a few peptidases and no cysteine peptidases at all were found therein. Nevertheless, the association of CB constructs with the midbody supports their nuclear occurrence.

### Transport mechanisms and interaction with the nuclear matrix

The exclusion limit for free diffusing molecules through the nuclear pores is at ~60 kDa. By applying constructs well above the exclusion limit (~84 kDa) we ruled out passive transport across the nuclear pore complex with high probability. The integrity of the products was proved by immunoblotting and by FRET analysis. In the time-lapse experiments, we noticed a directional transport of granules from across the cells to the Golgi and into the nucleus without any delay at the nuclear envelope. These observations ask for a specific transport system to which the expression product might be hooked into.

Are the imported artificial CB variants possibly retained inside the nucleus because of a specific affinity to nuclear components? To answer this question, a comparative TPM-photobleaching approach was applied. The mobility of CB(SC)-EGFP and ECFP-CB(SC)-EYFP was analysed in living cells by continuous photobleaching and FRAP. We chose the freely diffusing EGFP [41] and the tightly chromatin-bound H2A-EGFP [42] as limiting controls and TIF1A-EGFP as further control with partial mobile and immobile fractions. In both technical variants of the approach, the EGFP measurements obeyed curve shapes characteristic of free diffusion (almost exclusively mobile fraction); in contrast, those of H2A-EGFP were typical for predominantly immobile molecules. Hence, both controls reacted as to be expected and in analogy to former studies [49,50]. The courses of the CB(SC) graphs reflect an intermediate status indicating low immobile and high mobile fractions evolving from limited diffusion inside the nucleus. From this we assume that the artificially truncated CB is able to associate with nuclear matrix components. This nuclear affinity might be transferred to the naturally truncated Δ^51^CB form. Chromatin as a conceivable partner of interaction could be excluded from considerations by an OPM double labelling experiment of cells using GFP-tagged histone H2A and CB(SC) inasmuch as no colocalization could be observed. The relatively high amount of mobile CB(SC) might depend on scarcity of interaction partners: we have to consider that the CB products are overexpressed, other than their possible counterparts.

### Cell viability

It was reported that the naturally truncated Δ^51^CB was directed to the mitochondria and that the cells died after fragmentation of the nucleus [25]; our observations confirm these findings. We suppose that nuclear targeting of Δ^51^CB might be overwhelmed by the present MTS.

Removal of this sequence in the artificially truncated Δ^72^CB and in further modified constructs results in their nuclear targeting and accumulation. Both overexpressed natural and artificial constructs lead to the same consequence, namely nuclear fragmentation and cell death. Neither we nor others [25] could prove that the induced cell death arises from apoptosis.

It was described that cell death can be preceded by a release of mature and active CB from the lysosomes and by the appearance of CB in the nucleus [7]. Our studies of the artificially truncated constructs support these observations. Any truncated forms of CB proved to have no regular CB enzymatic activity. A proper refolding of Δ^51^CB to an enzymatically active form was demonstrated under *in vitro *conditions [24]. However, one has also to take into consideration a different cleavage activity or functionality for the truncated variant(s) of CB. Such was recently found in case of truncated cathepsin L [28] and probably also cathepsin H [27].

The significance of results, which are obtained by overexpression, is often a contentious issue. Two arguments support the validity of our results: (i) Generally, expression levels of transfected cell populations diverge largely among individual cells. In case of CB(SC), the response to expression is severe and comprises also cells with obviously negligible expression. (ii) Time lapse video sequences demonstrate a granulation and a directed transport of CB(SC) to the nuclear region followed by fusion with the nucleus (see supplemental material).

## Conclusion

Naturally appearing variations of CB and other related enzymes exhibit changed physiological characteristics and function as metabolic regulators in different states of diseases. We examined the nature of truncated CB by mutational analysis of extrinsic CB forms combined with advanced fluorescence microscopy. According to our results, artificially truncated CB forms lacking the MTS accumulated within the cell nucleus by an active transport mechanism and revealed binding affinity to nuclear matrix compounds. The region responsible for nuclear targeting resides in the C-terminal part of the protein. A hierarchy of signals is discussed. Expression of artificially truncated CB affected the cell viability to a large extent. Emerging from this, one has to raise the question whether the traditional understanding of distinct CB populations in terms of "normal" and "aberrant" might be misleading and thus has got to be reconsidered.

## Methods

### Cell lines

Investigations were performed on LCLC-103H cells derived from a human large cell lung carcinoma (ATCC# CCL5, DSMZ# 384). In addition to the original classification of these cells, it was now found that they are point-mutated in the *p53 *gene leading to an inactive p53. In control experiments, additional cell lines were used: HeLa (ATCC# CCL2), HEK-293 (DSMZ# 305), Wi-38 (ATCC# CCL75), primary human microvascular endothelial cells (HDMEC), Hep-G2 (DSMZ# 180), COS-7 (ATCC# CRL1651), and MDCK (ATCC# CCL34). The cell lines were cultured according to the recommendations of the suppliers.

### Transfection procedure

For microscopical studies, the cells were seeded on 4.2 cm-diameter coverslips or on Lab-Tek^® ^Chambered Cover Glasses (Nunc, Wiesbaden, Germany) at a density of 10^4^cm^-2 ^and transfected 12–15 h later at ~70% confluence with ~150 ng of the appropriate plasmid DNA by FuGENE6™ (Roche Molecular Biochemicals) according to the supplier's instructions. In double transfection experiments, equal masses of DNA were applied.

### Fluorescence microscopy

Prior to observation, the coverslips with the transfected cells were mounted in perfusion chamber holders (PeCon, Erbach, Germany). The samples were observed 24–72 h post transfection at 34–36°C and 5% CO_2_.

#### Digital wide field microscopy (WFM)

Images were obtained by an Axiovert S100TV (Zeiss, Jena, Germany). It was equipped with long distance condenser and objectives (Neofluar^® ^10×/0.30 Ph, LD Apochromat^® ^20×/0.40 Ph, LD Apochromat^® ^40×/0.60 Ph, Fluar^® ^40×/1.30 Oil, Plan-Neofluar^® ^63×/1.25 Oil Ph, C-Apochromat^® ^40×/1.20 W, Plan-Apochromat^® ^63×/1.4 Oil), a CCD camera (Orca C4742-95, Hamamatsu Photonics, Hamamatsu, Japan), shutters, macro (Ludl Electronic Products Ltd., Hawthorne, NY, USA) and piezo (Pifoc720, PI, Karlsruhe, Germany) focus drives, and an incubator to guarantee proper growth conditions in long-term experiments. The automated filter wheels (Ludl) contained filters for ECFP (Ex 436/10, DiM 455, Em 480/40), EGFP (Ex 488/20, DiM 505, Em 535/40), EYFP (Ex 515/10, DiM 530, Em 560/40) (Chroma, Brattleboro, VT, USA), and Cresyl Violet (Ex 560/40; DiM 590; Em 600 LP) (Omega Optical Inc., Brattleboro, VT, USA) fluorescence detection. Image acquisition was controlled by the OpenLab software (Improvision, Coventry, UK). Optical slices were partly subjected to built-in deconvolution algorithms and processed to 3D-restoration by the Amira™ software (TGS Europe, Düsseldorf, Germany).

#### TPM

Single scans and serial images for time-lapses were acquired by an Eclipse TE300 microscope (Nikon, Düsseldorf, Germany) using a Plan-Apochromat^® ^60×/1.2 W objective (Nikon). A pulsed (13.2 ns) mode-locked Mira900-F Ti:sapphire laser (Coherent, Santa Clara, CA, USA) was pumped by a Verdi™ argon laser (Coherent). TPM was performed at 860 nm; fluorescence was detected consecutively at 470/30 nm (ECFP), at 535/30 nm (EYFP), or at 510/20 nm (EGFP), respectively. Microscopical set-up and image processing were described in detail previously [51].

### Bleaching experiments

Nucleoplasmic diffusion of the fluorescent protein chimeras was analysed by photobleaching of living cells as described in [49] and shown exemplarily for the H2A-EGFP construct (Fig 5A). Besides minor modifications to the original work, which had to be introduced for technical reasons and which are described below in detail, TPM was used instead of OPM.

#### Fluorescence recovery after photobleaching (FRAP)

A nuclear region of 21.8 × 21.8 *μ*m^2 ^was scanned to describe the situation before bleaching (total scanning time: 2.033s; period duration: 5 *μ*s). The fluorescence was monitored at the respective emission wavelengths (see above) with 5 mW incident laser power. No cell damage was observed with the chosen illumination parameters. Within this area, a smaller region of interest (ROI) of 2 × 2 *μ*m^2 ^was bleached completely by 10 continuous scans (total bleaching time: 20.33 s). The selection was reset manually to the region at the beginning and a series of 50 consecutive single scans was recorded; the delay between bleaching and start of recording was ~2 s. About 8% of the initial EGFP fluorescence and up to 30% of the ECFP or EYFP intensity were depleted by the additional post-bleaching irradiation. Mean grey values of the images were evaluated by the Scion Image software (Scion Corporation; Las Vegas, NV, USA). The relative fluorescence intensity values, *I*_*rel*_, were normalized according equation (1)



where *T*_0 _is the total intensity before bleaching, *T*_*t *_the total intensity at various time points *t*, *I*_0 _the intensity of the prebleached ROI, and *I*_*t *_the intensity of the ROI at the corresponding time points. The share of the mobile fraction, *F*_*m*_, was calculated by equation (2)



where *I*_∞ _is the fluorescence in the ROI reaching a plateau after complete recovery, *I*_*i *_the intensity during prebleach and *I*_*b *_the intrinsic background fluorescence, which was negligibly low in our case. The effective diffusion coefficient, *D*_*eff*_, is conversely proportional to the characteristic diffusion time, *τ*_*D*_, which can be determined from the time value at *I*_∞_/2. For a more complex view of the calculations refer to [52,53].

#### Continuous photobleaching

The same basic parameters and evaluation tools as described for FRAP were used. A series of 30 consecutive images was grabbed within a square ROI of 2 *μ*m border width. Overview scans before and after the series were taken for comparison. The grey values of the ROI were normalized to their respective initial value and fitted by a regression function which was composed of two partial exponential terms by the SigmaPlot software (Systat Software, Inc., Point Richmond, CA, USA).

### Determination of the mortality rates

Mortality of transiently transfected LCLC-103H cell populations was quantified after a propidium iodide (Sigma-Aldrich, Taufkirchen, Germany) staining by a FACSCalibur™ flow cytometer (Becton-Dickinson, Heidelberg, Germany). The cell concentration was 2–8 × 10^5 ^cellsml^-1 ^in a measure volume of 50 *μ*l. Efficiency of transfection was determined by EGFP fluorescence in the FITC channel; dead cells stained by propidium iodide were monitored in the propidium iodide channel (excitation at 488nm each). The data were evaluated by the EPICS^® ^profile analyser software (Coulter Corp., Hialeah, FL, USA).

### Apoptosis tests

LCLC-103H cells transiently expressing CB(SC)-EGFP were checked for plasma membrane integrity and DNA fragmentation as indicators for apoptosis.

Plasma membrane perforation: At the cellular level, early stage apoptosis was examined by the AnnexinV-assay as described by the supplier (Roche Molecular Biochemicals, Mannheim, Germany). Cells were stained by AnnexinV and propidium iodide and subjected to fluorescence microscopy and FACS analysis.

DNA fragmentation: Genomic DNA was isolated and cleared of RNA using the DNA ladder kit (Roche Diagnostics, Mannheim, Germany) according to the instructions. Apoptosis was triggered by 5 *μ*m etoposide (Sigma-Aldrich) or MG115 (Sigma-Aldrich) in control cells. The samples were then subjected to agarose gelelectrophoresis.

### Cytochemistry

Besides GFP, synthetic living cell markers and immunolabels were used.

#### Living cell staining

Nuclear DNA was stained with 2 *μ*gml^-1 ^Hoechst33342 (Sigma-Aldrich), lysosomes were marked with 50 nM LysoTracker Red (Molecular Probes, Leiden, The Netherlands).

#### Immunolabelling

Methanol fixed cells immobilized onto poly-lysine coated cover slips were treated with antibodies (Abs) against CB (primary Ab: *α*-hCB-shIgG, BioAss, Herrsching, Germany; secondary Ab: *α*-sh-dIgG(HC+LC)-Cy3, Dianova, Hamburg, Germany), GFP (primary Ab: *α*-GFP-rIgG, Clontech, Heidelberg, Germany; secondary Ab: *α*-r-gIgG(HC+LC)-Cy3, Dianova), and the myc-epitope (primary Ab: *α*-myc-mIgG; secondary Ab: *α*-m-dIgG-Texas Red). Finally, the cover slips were mounted onto slides with Mowiol (Calbiochem, Schwalbach, Germany).

### Determination of the enzymatic activity

CB-specific activity was determined *invivo *by digital widefield microscopy (WFM). The fluorogenic substrate (Z-Arg-Arg)_2_-cresyl violet was added into the culture medium at 25 *μ*m and the following enzymatic reaction was monitored by the release of the fluorescent dye cresyl violet in 1 min intervals over a period of 10 min. For quantitative purposes, we used *invitro *activity assays, which were performed as described [54].

*SDS-PAGE and Western Blot analysis *– Proteins were separated on 12% polyacrylamide SDS gels followed by an electro transfer to a nitrocellulose membrane and detected by appropriate Abs using alkaline phosphatase by standard procedures.

### Cloning and mutational analysis

The sequence encoding the single chain form of human CB, CB(SC), was amplified from a known cDNA (IMAGE clone ID 380482; Deutsches Ressourcenzentrum für Genomforschung, Berlin, Germany) by PCR. It was subcloned into pcDNA3 (Invitrogen; NV Leek, NL) and tagged by appropriate fluorescent proteins (Clontech) analogously to the CB(FLM) sequence as described previously [55]. The CB(SC) sequence served as template for further mutation variants. Additional ATG start codons ensured proper translation. The artificially truncated sequence Δ^72^CB appeared as a by-product in the cloning process of CB(FLM) caused by an internal *Kpn*I restriction site. Specific mutations (insertion, deletion, conversion) were introduced into the CB sequence by site-specific mutagenesis using standard PCR procedures. All constructs were verified by DNA sequencing.

Synthesized forward (fd) and reverse (rv) primers (Pharmacia; DKFZ) were used for PCR amplification of modified CB constructs. They contained restriction sites for cloning into pcDNA3 (underlined in the following primer sequences): SC-fd-*Kpn*I: GGGGTACCATGCTGCCTGCAAGCTTCGATG, myc-SC-fd-*Kpn*I: GGGGTACCATGGAGCAGAAGCTGATCTCCGAGGAGGACCTGCTGCCTGCAAGCTTCGATGCACGG, SC-X-rv-*Not*I: GGCGGCCGCTTAATCGGTGCGTGGAATTCCAGC, [ΔC'pro]SC-rv-*Sal*I: GGGTCGACGATCTTTTCCCAGTACTGATCG, LC-rv-*Sal*I: GGGTCGACATTGGTGTGGATGCAGATGCGG, HC-fd-*Kpn*I: GGGGTACCATGGTCAGCGTGGAGCTGTCGG, HC_N'_-rv-*Sal*I: GGGTCGACATTGTATCCGTAGTGCTTGTCC, HC_C'_-fd-*Kpn*I: GGGGTACCATGAATTCCTACAGCGTCTCCA, HC_C'1_-fd-*Kpn*I: GGGGTACCATGGAGATCTACAAAAACGGCC, HC_C'2_-fd-*Kpn*I: GGGGTACCATGGTGTATTCGGACTTCCTGC, HC_C'3_-fd-*Kpn*I: GGGGTACCATGCAACACGTCACCGGAGAGA, HC_C'4_-fd-*Kpn*I: GGGGTACCATGCGCATCCTGGGCTGGGGAG, [E273L]SC-fd: CCAACACGTCACCGGACTGATGATGGGTGGCCATG, [E273L]SC-rv: CATGGCCACCCATCATCAGTCCGGTGACGTGTTGG, [E273del]SC-fd: ACCAACACGTCACCGGAATGATGGGTGGCCATGCC, [E273del]SC-rv: GGCATGGCCACCCATCATTCCGGTGACGTGTTGGT, [Q268_G277del]SC-fd: TACAAGTCAGGAGTGTACCATGCCATCCGCATCCTG, [Q268_G277del]SC-rv: CAGGATGCGGATGGCATGGTACACTCCTGACTTGTA, [L80_L283]SC-rv-*Sal*I: GGGTCGACCAGGATGCGGATGGCATGGCCA.

The spatial orientation of the mutated regions was visualized by the molecular modelling software PyMOL™ (v0.97, © DeLano Scientific LLC, San Carlo, California, USA). The reconstruction was based on crystallographic data of a mature CB protein (PDB-Id: *1huc*).

## List of abbreviations

4M*β*NA, 4-methoxy-*β*-naphthylamide; CB, cathepsinB; DSMZ, Deutsche Sammlung von Mikroorganismen und Zellkulturen GmbH; FLM, full length message; ECFP/EGFP/EYFP, enhanced cyan/green/yellow fluorescent protein; fd, forward; FP, fluorescent protein; FRAP, fluorescence recovery after photobleaching; FRET, fluorescence resonance energy transfer; HC, heavy chain; LC, light chain; MTS, mitochondrial targeting signal; NLS, nuclear localization signal; OPM, one-photon laser scanning microscopy; ℗, phosphate; ROI, region of interest; rv, reverse; SC, single chain; TIF1A, transcription initiation factor 1A; TPM, two-photon laser scanning microscopy; WFM, digital widefield microscopy; Z-Arg-Arg, benzyloxycarbonyl-Arginine-Arginyl

## Authors' contributions

FB carried out the molecular, biochemical, and microscopical studies, contributed to the cell culture, and drafted the manuscript. CD participated in the molecular cloning and the biochemical characterization, and contributed to the preparation of cells. ES carried out the cytochemical and enzymatic characterization of the cells, contributed to the microscopical studies, supervised the project, and drafted the manuscript. All authors have read and approved the final manuscript.

## Supplementary Material

Additional File 1 **Transient expression of CB(SC)-EYFP in LCLC-103H cells (phase contrast, fluorescence channel, and inverse grey value representation of the fluorescence signal). **The sequence describes the formation of granules, their fusion, and the transport to the nucleus accompanied by breakup of the Golgi. WFM, LD Apochromat^® ^40×/0.60 Ph. Δt = 5 min, total time = 195 min. Video format: MPEG1. The abbreviation "MM" (mature message) is used equivalently to the term "SC" (single chain). Click here for file

Additional File 2 **Transient expression of ECFP-CB(SC)-EYFP in LCLC-103H cells (fluorescence channels, phase contrast, and superimposition of the last fluorescence frames). **The double-tagged construct is expressed in the cytoplasm and accumulated within the nucleus. No significant differences among the two fluorescence channels can be observed suggesting that the polypeptide remains intact and is imported into the cell nucleus as a whole. Only the expressing cells undergo cell death and reveal membrane blebbing, which is typical for apoptotic processes. WFM; obj. Neofluar^® ^10×/0.30 Ph. Δt = 3 min, total time = 250 min. Video format: MPEG1. The abbreviation "MM" (mature message) is used equivalently to the term "SC" (single chain). Click here for file
